# A Novel Whole Gene Deletion of *BCKDHB* by Alu-Mediated Non-allelic Recombination in a Chinese Patient With Maple Syrup Urine Disease

**DOI:** 10.3389/fgene.2018.00145

**Published:** 2018-04-24

**Authors:** Gang Liu, Dingyuan Ma, Ping Hu, Wen Wang, Chunyu Luo, Yan Wang, Yun Sun, Jingjing Zhang, Tao Jiang, Zhengfeng Xu

**Affiliations:** ^1^State Key Laboratory of Reproductive Medicine, Department of Prenatal Diagnosis, The Affiliated Obstetrics and Gynecology Hospital of Nanjing Medical University, Nanjing Maternity and Child Health Care Hospital, Nanjing, China; ^2^Reproductive Genetic Center, Affiliated Hospital of Xuzhou Medical University, Xuzhou, China

**Keywords:** maple syrup urine disease, *BCKDHB*, dried blood spot, novel mutation, large deletion

## Abstract

Maple syrup urine disease (MSUD) is an autosomal recessive inherited metabolic disorder caused by mutations in the *BCKDHA, BCKDHB, DBT*, and *DLD* genes. Among the wide range of disease-causing mutations in *BCKDHB*, only one large deletion has been associated with MSUD. Compound heterozygous mutations in *BCKDHB* were identified in a Chinese patient with typical MSUD using next-generation sequencing, quantitative PCR, and array comparative genomic hybridization. One allele presented a missense mutation (c.391G > A), while the other allele had a large deletion; both were inherited from the patient’s unaffected parents. The deletion breakpoints were characterized using long-range PCR and sequencing. A novel 383,556 bp deletion (chr6: g.80811266_81194921del) was determined, which encompassed the entire *BCKDHB* gene. The junction site of the deletion was localized within a homologous sequence in two AluYa5 elements. Hence, Alu-mediated non-allelic homologous recombination is speculated as the mutational event underlying the large deletion. In summary, this study reports a recombination mechanism in the *BCKDHB* gene causing a whole gene deletion in a newborn with MSUD.

## Introduction

Maple syrup urine disease (MSUD, OMIM #248600) is an autosomal recessive inherited metabolic disorder, caused by a defective activity of the branched-chain α-keto acid dehydrogenase complex (BCKAD) complex in the mitochondria (Chuang et al., 2004). MSUD was first reported by Menkes et al. (1954). The worldwide incidence of MSUD is estimated to be 1 in 185,000 live births; however, in consanguineous ethnic groups, such as the Mennonite population, the incidence is as high as 1 in 380 live births (Nellis et al., 2003; Puffenberger, 2003; Chuang et al., 2006). BCKAD, a multimeric mitochondrial enzyme complex, is composed of multiple subunits of the branched-chain α-keto acid decarboxylase (E1), dihydrolipoamide branched-chain transacylase (E2), and dihydrolipoyl dehydrogenase (E3) (AEvarsson et al., 2000). E1 subunit shows a heterotetrameric structure consisting of two α subunits (E1α) and two β subunits (E1β), encoded by the *BCKDHA* and *BCKDHB* genes, respectively. E2 and E3 are encoded by the *DBT* and *DLD* genes, respectively. Pathogenic homozygous or compound heterozygous variants in *BCKDHA, BCKDHB, DBT*, or *DLD*, can lead to MSUD. BCKAD catalyzes the oxidative decarboxylation of branched-chain α-keto acids that are derived from the branched-chain amino acids (BCAAs), leucine, isoleucine, and valine. The deficient activity of BCKAD leads to the accumulation of leucine, isoleucine, valine and their respective metabolites causing systemic toxicity, but especially in the central nervous system (Yudkoff et al., 2005; Lang et al., 2010). The clinical presentation of the disease, which may include fatal ketoacidosis, neurological impairment and mental retardation, is dependent on the BCKAD residual activity. Based on the age of onset, clinical manifestation, and BCKAD residual activity, MSUD is divided into five types: classic, intermediate, intermittent, thiamine-responsive, and E3-deficient (Blackburn et al., 2017). The classic type with <2% of the normal enzyme activity exhibits the most severe phenotype. In the absence of timely diagnosis and treatment, the neonate with classic MSUD may succumb to early death due to brain edema from leucine neurotoxicity soon after birth (Levin et al., 1993; Strauss et al., 2010; Myers et al., 2012). In the present study, we described a Chinese patient with the severe classic form of MSUD caused by a missense mutation and a novel large deletion mutation in the *BCKDHB* gene.

## Materials and Methods

### Subjects

The male patient was diagnosed with MSUD based on the typical biochemical signs and eventually death at 20 days after birth. After 3 years, his parents visited the Nanjing Maternity and Child Health Care Hospital for preconception genetic counseling. Written informed consent was obtained from the parents for the molecular genetic analysis of the related genes and the publication of this case report, and this study was approved by the Ethics Committee of the hospital.

### DNA Extraction

Approximately, 3–5 mL peripheral blood samples were collected in EDTA anticoagulant tubes from the parents. Genomic DNA was prepared from blood specimens using a Simple^®^ Genomic DNA Whole Blood Kit and Simple^®^ Super Automated Nucleic Acid Extractor (Concert Bioscience, Xiamen, China) according to the manufacturer’s instructions. For the proband, the newborn dried blood spots filter paper was obtained from local Neonatal Screening Center. 3-mm diameter disks were punched, and the corresponding genomic DNA was prepared using Simple^®^ Genomic DNA Forensic Kit and Simple^®^ Super Automated Nucleic Acid Extractor (Concert Bioscience) according to the manufacturer’s instructions. The purified DNA was stored in TE buffer (10 mM Tris-HCl, pH 8.5) for downstream applications.

### Next-Generation Sequencing

Molecular analysis of *BCKDHA, BCKDHB, DBT*, and *DLD* genes in both parents of the patient was performed using targeted next-generation sequencing on Ion Torrent Personal Genome Machine platform (Life Technologies, Waltham, United States). The genomic DNA samples were used for PCR enrichment of target genes using Ion AmpliSeq Custom Panel (Life Technologies). The amplification library of the target exons was prepared using an Ion Ampliseq Library Kit 2.0 and Ion Xpress Barcode Adapter 1-16 Kit (Life Technologies). The emulsion PCR was performed using the Ion OneTouch^TM^ System and Ion PGM^TM^ Hi-Q^TM^ View OT2 Kit (Life Technologies). Then, template-positive Ion Sphere^TM^ particles were enriched using the Dynabeads^®^ MyOne^TM^ Streptavidin C1 Beads and washed with the Ion OneTouch Wash Solution included in the kit using an Ion OneTouch ES system (Life Technologies). A parallel DNA sequencing was performed with a PGM system using the Ion PGM^TM^ Hi-Q^TM^ View Sequencing Kit and Ion 318^TM^ Chip v2 (Life Technologies) according to the manufacturer’s instructions. The sequencing data were processed with standard Ion Torrent Suite^TM^ Software 4.2 utilizing the reference human genome GRCh37/hg19 assembly. After variant calling, all the detected variants were filtered against dbSNP142. The sequencing results were visualized by the Integrated Genomics Viewer (Robinson et al., 2011). In addition, the candidate disease-causing variant in exon 4 of the *BCKDHB* gene was confirmed by PCR and Sanger direct sequencing: forward primer 5′-TCCTGGTCTCAAGTAATCCTCTT-3′ and reverse primer 5′-GTAGCCTTGGACTCCTGGTT-3′.

### Real-Time PCR

To detect the *BCKDHB* gene deletion/duplication, we developed a gene dosage assay based on real-time quantitative PCR (qPCR). Five pairs of primers targeting exons 1, 2, 4, 9, and 10 based on the reference sequence NM_000056.4 and NG_009775.1 were designed using Primer 3 software (Supplementary Table S1) (Ye et al., 2012). Real-time PCR was performed in a total volume of 20 μL consisting of 10 μL AceQ qPCR SYBR Green Master Mix (Vazyme), 0.4 μL of 50X ROX reference dye 1 (Vazyme, Nanjing, China), 0.4 μL of 10 μM each primer (forward and reverse), 2 μL genomic DNA diluted from 10 ng/μL, and water. The PCR reaction on StepOnePlus^TM^ Real-Time PCR System (Life Technologies) was as follows: an initial denaturation step (95°C for 5 min), followed by 40 cycles of two-step amplification and fluorescence detection (94°C for 10 s and 60°C for 30 s), and a melting curve in a single cycle of 95°C for 15 s, 60°C for 60 s, and 95°C for 15 s, while continuously measuring the change in fluorescence intensity. At the end of PCR, the 5 exon dosages of *BCKDHB* gene were calculated by the StepOne^TM^ Software using the comparative CT (ΔΔCT) quantitation method. The exon 4 of *ACTB* gene was used as the normal copy number reference.

### Array CGH Analysis

In order to detect the copy number variation (CNV) and deletion size, array comparative genomic hybridization (array CGH) was performed using the Affymetrix CytoScan^®^ 750K arrays (Affymetrix, Santa Clara, United States) according to the manufacturer’s instructions. The platform includes 550,000 unique non-polymorphic CNV probes and approximately 200,000 genotypable SNP probes with an average marker spacing of >4 kb. The genomic DNA digestion, ligation, fragmentation, labeling, hybridization, staining, and scanning were performed according to the manufacturer’s protocol. The data were analyzed by Chromosome Analysis Suite (ChAS) v3.1 Software (Affymetrix, Santa Clara, USA), and the February 2009 human reference sequence (GRCh37/Hg19) was used for genomic annotation.

### Long-Range PCR Analysis

To further identify the breakpoint junction of the gross deletion in the *BCKDHB* gene, a forward primer (5′-GTGTTTAGGAGTGGCTTTACATGGTGAGGAAATC-3′ corresponding to chr6:80809183-80809216) and a reverse primer (5′- GTTGAATGAACAAAGAAGGAACCACAGACAAGAGA-3′ corresponding to chr6:81197956-81197990), flanking the predicted breakpoints were designed using Primer 3 software based on the reference sequence chr6: 80,804,685- 81,214,051 from UCSC hg19 for long-range polymerase chain reaction (LR-PCR) (Ye et al., 2012). In this LR-PCR assay, the 50 μL PCR mixture contains 10 μL of 5X PrimeSTAR GXL Buffer, 8 μL of dNTP Mixture (2.5 mM each), 1 μL of PrimeSTAR GXL DNA Polymerase (Takara, Shiga, Japan), 1 μL of 10 μM each primer (forward and reverse), 5 μL betaine solution (5 M, PCR reagent) (Sigma-Aldrich, Seelze, Germany), 50 ng genomic DNA, and water. The LR-PCR was performed using 96-Well Veriti^®^ Thermal Cycler (Applied Biosystems, Foster City, United States) as follows: 1 min at 98°C for an initial denaturation step, followed by 30 amplification cycles at 98°C for 10 s, 68°C for 15 min, and 72°C for 15 min for a final extension step. The PCR products were resolved by 0.8% agarose gel electrophoresis GelRed (Biosharp, Hefei, China) in 1 × TAE buffer for 15 h at 15 mA, the followed by visualization using the Molecular Imager^®^ Gel Doc^TM^ XR System (Bio-Rad, CA, United States). After purification using a MultiScreen^®^ PCR96 Filter Plate (Millipore, Billerica, United States), the purified products were sequenced on an ABI 3730xl DNA automated sequencer (Applied Biosystems). The contig assembly of the raw data obtained by Sanger sequencing was performed using the SeqMan module of Lasergene software (DNASTAR, Madison, United States).

### Bioinformatics Analysis

Approximately, 5000 bp of the contig sequence from LR-PCR was analyzed to identify two breakpoints of the gross deletion with BLAT using the UCSC Genome Browser^1^. The known repetitive elements were evaluated by CENSOR^2^ at the 300 nucleotide sequence flanking each breakpoint using the February 2009 human reference sequence (GRCh37/hg19) (Kohany et al., 2006).

## Results

### Characteristics of the Proband

The proband was the first child born to healthy, non-consanguineous parents after an uneventful pregnancy. The child was born at 39 weeks of gestation by cesarean section. He developed feeding difficulties, seizure, and lethargy at 10 days of age; and was referred to a local hospital immediately. Maple syrup-like urine was observed, and tandem mass spectrometry (MS/MS) using dried blood spots showed 7172.76 μmol/L leucine (normal range 93∼365 μmol/L), 783.72 μmol/L valine (normal range 81∼307 μmol/L), and the rate of leucine/phenylalanine as 53.82 (normal range 1.5∼5.8). The patient was diagnosed with MSUD, and he deceased at the age of 20 days. After 3 years, the parents were referred to our clinic for preconception genetic counseling when they expressed concern about the recurrent risk of the disorder in future offspring. Therefore, the parents underwent molecular genetic analysis to identify the genetic cause of MSUD in the family.

### Molecular Analysis

**FIGURE 1:**
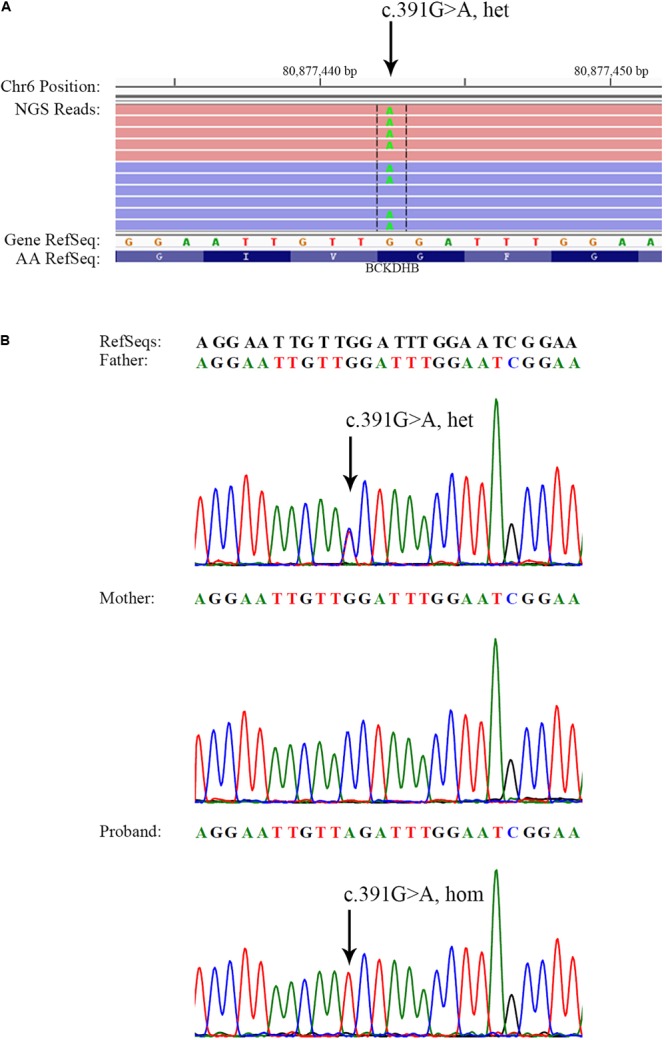
Identification of a *BCKDHB* c.391G > A mutation in the father and the proband. **(A)** Visualization of c.391G > A in the *BCKDHB* gene as heterozygous in the father of the proband. NGS reads were piled and are shown on the Integrative Genomics Viewer. **(B)** Sanger sequencing showing the *BCKDHB* c.391G > A mutation in the father and the proband. The same mutation was not found in the *BCKDHB* gene of the mother. Black arrows indicate the point mutation.

Next-generation sequencing of the exons of *BCKDHA* (NM_000709.3), *BCKDHB* (NM_000056.4), *DBT* (NM_001918.3), and *DLD* (NM_000108.4) genes revealed a heterozygous mutation c.391G > A in the *BCKDHB* gene of the proband’s father, which resulted in the conversion of glycine to arginine at the amino acid position 131 (p.Gly131Arg) (**Table 1** and **Figure 1**). This mutation has been reported as a causative mutation of MSUD (Cheng et al., 2017). No reported or suspicious mutations were identified in the genetic profile of the proband’s mother; nonetheless, two homozygous variations of the *BCKDHB* gene were revealed (**Table 1**).

**Table 1:** The variations in *BCKDHA, BCKDHB, DBT*, and *DLD* genes detected in the parents.

Sample	Gene	Chromosome coordinate	Nucleotide change	Amino acid change	Type	MAF	dbSNP ID
Father	*BCKDHA*	chr19:41903675	c.-58A>G	–	Homozygous	0.0202	rs892043
	*BCKDHB*	chr6:80837239	c.197-25A>G	–	Homozygous	0.3878	rs9448893
	*BCKDHB*	chr6:80877442	c.391G>A	p.Gly131Arg	Heterozygous	–	–
	*BCKDHB*	chr6:80982701	c.952-151G>A	–	Homozygous	0.4026	rs9341811
	*BCKDHB*	chr6:81053217	c.1039-164G>T	–	Homozygous	0.2859	rs2322763
	*DBT*	chr1:100715454	c.-78C>T	–	Homozygous	0.2750	rs3806235
	*DLD*	chr7:107531279	c.-417A>T	–	Heterozygous	0.2760	rs6943999
	*DLD*	chr7:107545586	c.438+83G>A	–	Heterozygous	0.2758	rs17412104
	*DLD*	chr7:107545799	c.439-7T>C	–	Heterozygous	0.2840	rs10263341
	*DLD*	chr7:107559722	c.^∗^18A>T	–	Heterozygous	0.2758	rs8721
Mother	*BCKDHA*	chr19:41919944	c.376-10A>C	–	Homozygous	0.3840	rs3213861
	*BCKDHA*	chr19:41928652	c.972C>T	p.Phe324 =	Homozygous	0.3836	rs284652
	*BCKDHA*	chr19:41928765	c.995+90C>T	-	Homozygous	0.3860	rs284655
	*BCKDHA*	chr19:41930396	c.1221A>G	p.Leu407 =	Homozygous	0.3830	rs4674
	*BCKDHA*	chr19:41903699	c.-34T>G	–	Heterozygous	0.0825	rs45500792
	*BCKDHB*	chr6:80877371	c.344-24C>T	–	Homozygous	0.1298	rs73479953
	*BCKDHB*	chr6:80912977	c.951+48C>T	–	Homozygous	0.1296	rs3749896
	*DBT*	chr1:100715454	c.-78C>T	–	Homozygous	0.2750	rs3806235
	*DLD*	chr7:107531203	c.-493C>T	–	Homozygous	0.3636	rs34572011
	*DLD*	chr7:107545799	c.439-7T>C	–	Homozygous	0.2840	rs10263341
	*DLD*	chr7:107559732	c.^∗^28G>T	–	Homozygous	0.2003	rs17154615

*(1) Chromosome coordinates were based on the NCBI37/hg19 assembly of the human genome. NM_000709.3, NM_000056.4, NM_001918.3, and NM_000108.4 were employed as reference sequences for *BCKDHA, BCKDHB, DBT*, and *DLD*, respectively. (2) The symbol “–” indicates no change or record and the symbol “ = ” indicates a synonymous mutation or no change in amino acid residues. (3) “MAF,” referred to the frequency of the minor allele in 1000 Genomes database. (4) “dbSNP ID,” referred to the identification number of variations in NCBI dbSNP database 142.*

**FIGURE 2:**
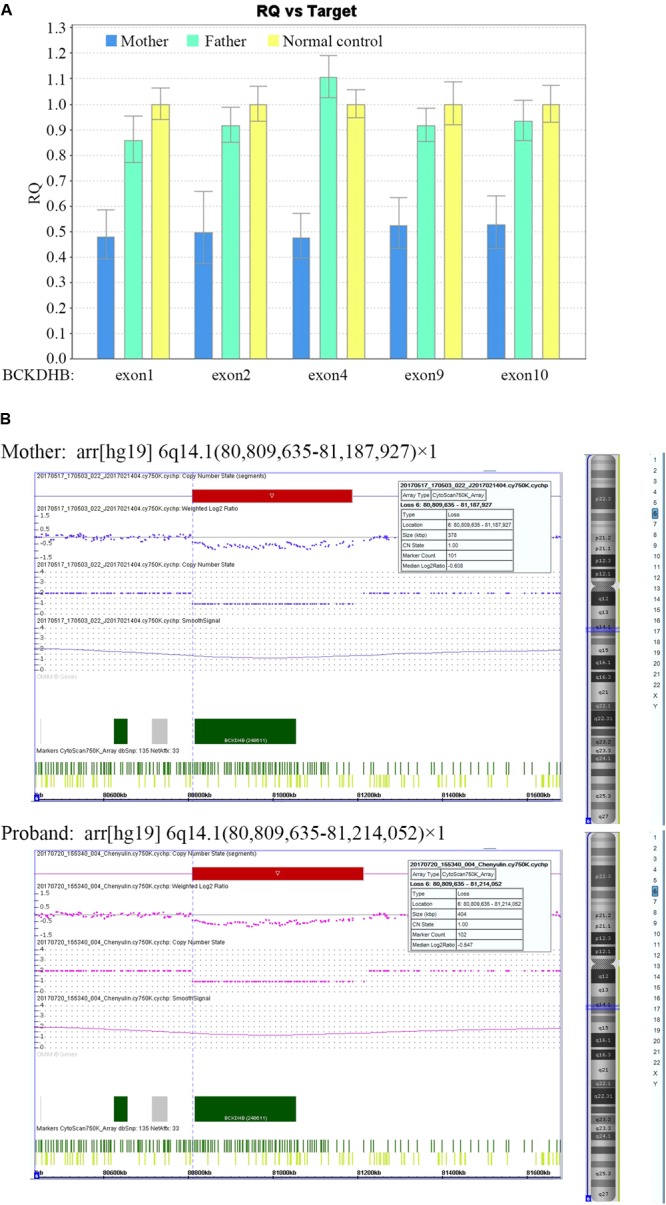
Identification of a large deletion mutation in *BCKDHB* gene in the mother and the proband. **(A)** The gene dosage assay based on qPCR. The exon copy numbers of the father and the mother were normalized against the normal control. The mean dosage ratios of exons 1, 2, 4, 9, and 10 in the *BCKDHB* gene of the mother were approximately 0.5, suggesting heterozygous deletions. **(B)** CNV analysis of the mother and the proband using Affymetrix CytoScan 750K microarray platform. A ∼378 kb gross deletion of arr[hg19] 6q14.1(80,809,635-81,187,927) × 1 was detected in the mother, while a ∼404 kb gross deletion of arr[hg19] 6q14.1(80,809,635-81,214,052) × 1 was detected in the proband. The two deletions encompass the whole *BCKDHB* gene.

The gene dosage assay revealed that the father harbored the two normal copies of exons 1, 2, 4, 9, and 10 of *BCKDHB*, whereas the mother had only one copy of these exons (**Figure 2A**), which indicated that the mother carried a large deletion in the *BCKDHB* gene. Moreover, the array CGH analysis revealed that a ∼378 kb gross deletion of arr[hg19] 6q14.1(80,809,635-81,187,927) × 1 was present in the mother and a ∼404 kb gross deletion of arr[hg19] 6q14.1(80,809,635-81,214,052) × 1 was present in the proband (**Figure 2B**). The deletion region contained only one OMIM gene: *BCKDHB*.

**FIGURE 3:**
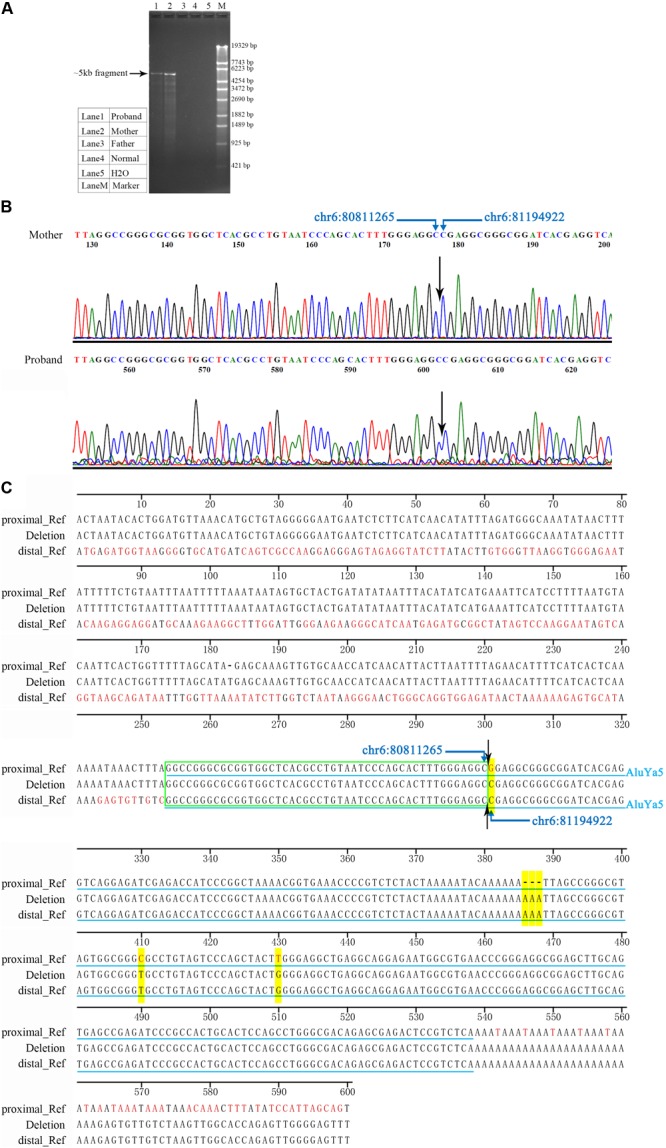
Characterization of the junction site of the deletion. **(A)** Electropherogram of LR-PCR products for the identification of the deletion breakpoints. In the LR-PCR analysis, the proband and the mother presented ∼5 kb fragments, whereas the father and a normal control lacked such fragments. Lane 1, the proband. Lane 2, the proband’s mother. Lane 3, the proband’s father. Lane 4, a normal control DNA sample. Lane 5, blank control H2O. Lane M, DNA marker λ-EcoT14 I digest. **(B)** Sanger electropherogram is showing the breakpoint junction of the deletion. Sanger sequences from the mother were identical to that of the proband at the single nucleotide level. The black arrow indicated the breakpoint junction. **(C)** Sequence comparison between the deletion junction at the proximal and distal breakpoints. Approximately, 600-bp sequence surrounding each breakpoint was analyzed. Two Alu elements (AluYa5) were identified and highlighted by light blue lines. Yellow highlight indicated different nucleotides between the two AluYa5 elements. Symbol “–” indicated no nucleotide base. Green wireframe indicated 47 bp same homologous sequence.

In order to further characterize the junction site of the large deletion, LR-PCR was performed, and the PCR products detected by agarose gel electrophoresis. Specimens from both the proband and the mother demonstrated the ∼5 kb fragments. None of the fragments were amplified in specimens from the father and normal control (**Figure 3A**). The normal size amplicons (expected size, 388,808 bp) were large and failed to display any bands under the PCR conditions. Thus, the ∼5 kb fragment was suggested as the result of a large deletion between the two primer sites of the LR-PCR primer pair. Both ∼5 kb fragments were subjected to Sanger sequencing (**Figure 3B**). The assembly sequence from the proband was identical to that from his mother. The sizes of the two fragments were same: 5153 bp. Using chromosome 6 contig NC_000006.11 as a reference, the proximal deletion breakpoint mapped somewhere between 80,811,219 and 80,811,265 region, while the distal deletion breakpoint was mapped between 81,194,875 and 81,194,921 position. The proximal and distal breakpoints were localized within the same homologous sequence with a size of 47 bp (**Figure 3C**). According to the mutation nomenclature recommended by HGVS, the large deletion was described as chr6: g.80811266_81194921del383556. The bioinformatics analysis of the 600-bp sequence surrounding each breakpoint was performed using the CENSOR program. An AluYa5 element was identified at both the proximal and distal breakpoint loci. The AluYa5 in the proximal breakpoint shared a 99.6% sequence similarity to the AluYa5 reference, and the AluYa5 in the distal breakpoint had a 98.9% sequence similarity. The recombination between the two AluYa5 elements resulted in a new hybrid AluYa5 element (**Figure 3C**).

## Discussion

The c.391G > A mutation in the *BCKDHB* gene has been reported to be responsible for MSUD in a Chinese patient (Cheng et al., 2017). The MSUD patient in this study was the second case carrying the mutation. Gly131 is a highly conserved amino in several species, and the substitution from polar the uncharged glycine to charged arginine proposed a damaging mutant with a score of 1.000 (sensitivity: 0.00; specificity: 1.00) using PolyPhen2 algorithm (Adzhubei et al., 2010). The key role of glycine in the structural stability of proteins has been described previously (Pakula and Sauer, 1989). Therefore, the amino acid residue Arg131 could affect the structure and function of the branched-chain alpha-keto acid dehydrogenase complex, rendering the mutation pathogenic.

To date, only one deletion in the *BCKDHB* gene has been identified, in an Iranian patient with a homozygous deletion of exon 3 in the *BCKDHB* gene (Abiri et al., 2017). However, the deletion has not yet been well characterized by another method, and the deletion breakpoints are unknown. Such a large deletion, also known as a copy number variant (CNV), arise from different mutation mechanisms than do point mutations and small insertions and deletions (indels), such as non-allelic homologous recombination (NAHR), non-homologous end joining (NHEJ), and fork stalling and template switching/microhomology-mediated break-induced replication (FoSTeS/MMBIR) (Gu et al., 2008; Lieber, 2008; Zhang et al., 2009; Liu et al., 2011). The current study describes the second large deletion in the *BCKDHB* gene, thereby providing insights into the mechanisms underlying the genomic rearrangement. The two AluYa5 elements, belonging to the short interspersed elements (SINEs), are localized in the two breakpoints of the large deletion (Mamedov et al., 2005). Alu elements are frequently involved in genomic rearrangements including deletion, because of its frequency of 1.1 million copy numbers in the human genome with a high degree of sequence homology. In addition, these elements are associated with roughly 0.1% of the human genetic disorders (Kim et al., 2016). Moreover, two low-copy repeats or repetitive sequences such as LINE and Alu elements can drive NAHR events leading to genomic rearrangements such as recurrent insertions and deletions with similar sequence sizes and clustered breakpoints (Shaw and Lupski, 2004; Lee and Lupski, 2006; Gu et al., 2008; Carvalho and Lupski, 2016).

The comparison of the two AluYa5 elements involved in this deletion with the newly formed AluYa5 element that results from the deletion, revealed that the junction was localized within a homologous sequence in both AluYa5. Therefore, Alu-mediated NAHR was a putative mutational event underlying the large deletion. Furthermore, about 80 Alu elements are distributed throughout the entire *BCKDHB* gene, creating DNA sequence contexts prone to mutation events. AluYa5 is the most active Alu element in the human lineage (Konkel et al., 2015). Only two AluYa5 elements exist in the *BCKDHB* gene (upstream and downstream), the ones involved in the large deletion observed in our patient.

In this study, we identified compound heterozygous *BCKDHB* mutations in our patient: a missense mutation (c.391G > A) and a large deletion mutation (chr6: g.80811266_81194921del383556). The current results provided accurate information for genetic counseling, including the possibility of prenatal and preimplantation diagnosis. The analysis of the junctional fragments revealed that the two AluYa5 elements were located at the proximal and distal breakpoint loci, respectively. This finding suggests that Alu-mediated NAHR may be the mechanism underlying the identified large deletion. Also, we emphasize that large deletions in the *BCKDHB* gene should be considered if a molecular diagnosis cannot be achieved through routine methods.

## Author Contributions

GL and DM performed the molecular studies, analyzed the data, and wrote the manuscript. DM, PH, and WW reviewed all *BCKDHB* mutations in the literature. CL, YW, YS, and JZ collected clinical data and performed clinical evaluation. TJ and ZX designed the study and reviewed the manuscript.

## Conflict of Interest Statement

The authors declare that the research was conducted in the absence of any commercial or financial relationships that could be construed as a potential conflict of interest.

## Footnotes

1 http://www.genome.ucsc.edu

2 http://www.girinst.org/censor/index.php
