# Supplementary material for: A Novel Whole Gene Deletion of BCKDHB by Alu-Mediated Non-allelic Recombination in a Chinese Patient With Maple Syrup Urine Disease
Source: Front Genet. 2018 Apr 24;9:145. doi: 10.3389/fgene.2018.00145 (PMC5928131; doi:10.3389/fgene.2018.00145)
Supplement: Supplementary file 1 [file Table_1.DOCX]

**Table S1. qPCR primers used in the detection of exons of the *BCKDHB* gene**

| **Targets** | **Exon** | **Primer name** | **Sequence (5’-3’)** | **Tm^c^** | **Product length** |
| --- | --- | --- | --- | --- | --- |
| *BCKDHB*^a^ | 1 | ex1QF | AGTTCCGATTGGTCTGTTTCAT | 60 ℃ | 116 bp |
|  |  | ex1QR | CGGGATTCTCAGGCTATGC |  |  |
|  | 2 | ex2QF | TTCACAGGGCAAACTCAGAAA | 60 ℃ | 117 bp |
|  |  | ex2QR | GCTACCACAATTCAGGCACATAT |  |  |
|  | 4 | ex4QF | ACTCTCATTTGCCACATTAACC | 60 ℃ | 115 bp |
|  |  | ex4QR | GACCGCAATTCCGATTCCA |  |  |
|  | 9 | ex9QF | TCACGAGGCTCCCTTGACA | 60 ℃ | 116 bp |
|  |  | ex9QR | CTTCTGGAATTGGCATGTGGAA |  |  |
|  | 10 | ex10QF | TCTTGAACCTAGAGGCTCCTATATC | 60 ℃ | 108 bp |
|  |  | ex10QR | GGGCATCATAACACTTCCATTTG |  |  |
| *ACTB*^b^ | 4 | ex4QF | GCCATGTACGTTGCTATCCA | 60 ℃ | 112 bp |
|  |  | ex4QR | CCTCGTAGATGGGCACAGT |  |  |

Notes: ^a^ Based on reference sequence NM_000056.4 and NG_009775.1; ^b^ Based on the reference sequence, NM_001101.4 and NG_007992.1; ^c^ Tm, the annealing temperature of the PCR product.
